# Discharge Clinical Characteristics and Post-Discharge Events in Patients with Severe COVID-19: A Descriptive Case Series

**DOI:** 10.1007/s11606-020-06494-7

**Published:** 2021-02-02

**Authors:** Faysal G. Saab, Jeffrey N. Chiang, Rachel Brook, Paul C. Adamson, Jennifer A. Fulcher, Eran Halperin, Vladimir Manuel, David Goodman-Meza

**Affiliations:** 1 David Geffen School of Medicine, Ronald Reagan UCLA Medical Center, 757 Westwood Blvd., Suite 7501, Los Angeles, CA 90095 USA; 2 Department of Computational Medicine, UCLA, California, Los Angeles USA; 3 Division of Infectious Diseases, David Geffen School of Medicine, UCLA, Los Angeles, CA USA; 4 UCLA Clinical and Translational Science Institute, Los Angeles, CA USA; 5 Department of Computer Science, UCLA, Los Angeles, CA USA; 6 Department of Anesthesiology and Perioperative Medicine, UCLA, Los Angeles, CA USA; 7 Department of Human Genetics, UCLA, Los Angeles, CA USA; 8 Institute of Precision Health, UCLA, Los Angeles, CA USA; 9 Faculty Practice Group, David Geffen School of Medicine, UCLA, Los Angeles, CA USA

**Keywords:** COVID-19, discharge, inflammatory markers, fever, oxygen

## Abstract

**Background:**

As the SARS-CoV-2 pandemic continues, little guidance is available on clinical indicators for safely discharging patients with severe COVID-19.

**Objective:**

To describe the clinical courses of adult patients admitted for COVID-19 and identify associations between inpatient clinical features and post-discharge need for acute care.

**Design:**

Retrospective chart reviews were performed to record laboratory values, temperature, and oxygen requirements of 99 adult inpatients with COVID-19. Those variables were used to predict emergency department (ED) visit or readmission within 30 days post-discharge.

**Patients (or Participants):**

Age ≥ 18 years, first hospitalization for COVID-19, admitted between March 1 and May 2, 2020, at University of California, Los Angeles (UCLA) Medical Center, managed by an inpatient medicine service.

**Main Measures:**

Ferritin, C-reactive protein, lactate dehydrogenase, D-dimer, procalcitonin, white blood cell count, absolute lymphocyte count, temperature, and oxygen requirement were noted.

**Key Results:**

Of 99 patients, five required ED admission within 30 days, and another five required readmission. Fever within 24 h of discharge, oxygen requirement, and laboratory abnormalities were not associated with need for ED visit or readmission within 30 days of discharge after admission for COVID-19.

**Conclusion:**

Our data suggest that neither persistent fever, oxygen requirement, nor laboratory marker derangement was associated with need for acute care in the 30-day period after discharge for severe COVID-19. These findings suggest that physicians need not await the normalization of laboratory markers, resolution of fever, or discontinuation of oxygen prior to discharging a stable or improving patient with COVID-19.

## INTRODUCTION

At the time of this submission, SARS-CoV-2, the virus causing coronavirus disease 2019 (COVID-19), has infected over 38 million people and claimed over one million lives worldwide.^1^ In many densely populated regions, it has overwhelmed healthcare facility capacity and caused widespread medical supply shortages. The novelty of this virus has spurred record-breaking efforts by the scientific community to describe its epidemiology, illustrate its clinical features, develop therapeutics, and build illness severity prediction tools to aid clinicians in its triage and management.

From those efforts, we have learned that SARS-CoV-2 may cause immune dysregulation highlighted by elevations in inflammatory markers such as ferritin, C-reactive protein (CRP), D-dimer, and lactate dehydrogenase (LDH).^2–4^ Profound lymphopenia, elevated procalcitonin, and mild transaminitis are also common features.^3–5^ White blood cell count (WBC), absolute lymphocyte count (ALC), and CRP have been described as risk factors for disease progression.^6^ Numerous studies have correlated such laboratory derangements with disease severity, including need for intensive care, development of acute respiratory distress syndrome (ARDS), and death.^7–15^

However, it remains unclear how laboratory results might be used to inform medical management in patients with severe COVID-19, defined as SpO_2_ < 94% on room air at sea level, a ratio of arterial partial pressure of oxygen to fraction of inspired oxygen (PaO_2_/FiO_2_) < 300 mmHg, respiratory rate > 30 breaths/min, or lung infiltrates > 50% of lung fields.^16^ Many of these hospitalized patients have persistently elevated inflammatory markers and, given variable plasma half-lives, some markers may remain elevated despite clinical improvement.^17^ Also unknown is how other inpatient indices, such as persistent fever and oxygen requirement, inform discharge readiness in otherwise stable or improving patients. The Centers for Disease Control (CDC), in addition to various medical centers, have suggested specific COVID-19 discharge criteria, recommending absence of fever for 24–48 hours and stable inflammatory markers.^18^ These guidelines likely reflect clinical practice habits rather than an existing body of evidence.

Healthcare facilities worldwide are facing inpatient bed shortages, causing clinicians to make difficult discharge decisions in the face of persistent clinical abnormalities. In the absence of constraints on healthcare resources, patients might be monitored for resolution of fever and laboratory markers further trended until the clinician and patient feel that a discharge is safe. Yet, in these pressing times, urgent clinical guidance based on outcomes data is needed to inform shared decision-making about patient discharge.

Here, we aimed to describe the peri- and post-discharge courses of patients with severe COVID-19. We evaluated whether abnormal laboratory results, persistent fever, or oxygen requirement around the time of discharge were associated with repeat emergency department (ED) visits or readmissions. Clarifying the significance of these features could aid clinical decision-making as it pertains to hospitalized patients with COVID-19.

## METHODS

We performed a retrospective chart review of 147 patients hospitalized at UCLA Medical Center between March 1, 2020, and May 2, 2020. Patients were identified using an internal registry. COVID-19 was the primary reason for hospitalization and patients were managed by an inpatient medicine service. We excluded patients who were pregnant, < 18 years old, those incidentally found to be COVID-PCR positive, and those who were deceased during the hospitalization. The retrospective chart review was approved by the UCLA institutional review board (20-00473).

For each patient, post-discharge documentation in the form of a clinical telephone or telehealth video note in the electronic health record was assessed by two hospitalists involved in the study (FS, RB). The following data were manually extracted: day of follow-up documentation, oxygen requirement at discharge, highest temperature within 24 h of discharge, post-hospitalization ED visit, and hospital readmission at UCLA or another facility whose electronic health record communicated with the UCLA Health System. These notes also mentioned whether the patient had clinically worsened and required any kind of acute care in the interim, facilitating capture of post-discharge events occurring outside of the UCLA Health System.

The sequential results of a complete blood count with differential, ferritin, D-dimer, lactate dehydrogenase, procalcitonin, and C-reactive protein, in addition to patient temperature and oxygen requirement, were extracted from the chart. Associations between the last recorded lab values and oxygen requirement prior to the time of discharge were tested for association with post-hospitalization ED visits and hospital readmission using a one-way ANCOVA with age and sex as covariates. Post hoc pairwise *T* tests using the Tukey HSD were also performed where indicated.

## RESULTS

In total, 147 patients were identified in the registry. Fifteen patients were deceased prior to discharge; 16 patients were not adults, not hospitalized primarily for COVID, or were managed by a non-medicine service, and were thus excluded. Two patients were discharged on hospice with expected death and were also excluded. Of the remaining 114 adult patients, 99 (86.8%) had 30-day follow-up documented in the electronic health record and were included in the analysis.

Patient clinical characteristics are described in Table 1. Three groups were analyzed: (1) patients without a COVID-related ED visit within 30 days of discharge; (2) patients with a potentially COVID-related ED visit within 30 days of discharge; and (3) patients with a potentially COVID-related readmission within 30 days of discharge. Of 99 patients, five required an ED admission within 30 days, and another five required readmission. The median length of follow-up was 42 days (range 30–86 days).

**Table 1:** Clinical Characteristics of Patients

	Total	No Post-Discharge Encounter	Post-Discharge ED visit	Readmission
Patients	99	89	5	5
Sex (no. (%))				
Male	64	58 (65.1)	3 (60)	3 (60)
Female	35	31 (34.9)	2 (40)	2 (40)
Mean age (SD)	59.8	59.6 (18.0)	59.9 (18.2)	64.6 (17.9)
Asian	11	10 (11.2)	1 (20)	0
Black	3	3 (3.3)	0	0
Latinx	32	28 (31.4)	3 (60)	1 (20)
White	39	35 (39.3)	1 (20)	3 (60)
Other	14	13 (14.6)	0	1 (20)
Mean length of stay-days (SD)	10.1	10.5 (9.9)	8.4 (5.4)	5 (3.5)
Mean days of symptoms at discharge (SD)	17.8	18.0 (10.1)	13.4 (6.8)	17.2 (7.2)
Required oxygen at discharge (no. (%))	31	29 (33)	1 (20)	1 (20)
Febrile within 24 h of discharge (no. (%))	4	4 (4.5)	0 (0)	0 (0)
Hypertension (no. (%))	49	43 (48.3)	3 (60)	3 (60)
Type 2 diabetes mellitus (no. (%))	27	25 (28)	1 (20)	1 (20)
End-stage renal disease (no. (%))	10	8 (9.0)	2 (40)	0 (0)
Chronic obstructive pulmonary disease (no. (%))	5	5 (5.6)	0 (0)	0 (0)
Asthma (no. (%))	9	8 (8.9)	1 (20)	0 (0)
Current smoker (no. (%))	2	2 (2.2)	0 (0)	0 (0)
Ramdesir (no. (%))	13	13 (14.6)	0 (0)	0 (0)
Glucocorticoids (no. (%))	12	10 (11.2)	1 (20)	1 (20)
Tocilizumab (no. (%))	15	15 (16.9)	0 (0)	1 (20)
Sarilumab (no. (%))	8	7 (7.9)	1 (20)	0 (0)
Leronlimab (no. (%))	18	15 (16.9)	3 (60)	0 (0)
Azithromycin (no. (%))	67	61 (68.5)	2 (40)	4 (80)
Immunosuppression (no. (%))	15	12 (13.4)	1 (20)	2 (40)
ACE inhibitor or ARB (no. (%))	22	19 (21.3)	1 (20)	2 (40)

Eighteen of 99 patients (18.18%) had a post-discharge ED visit during this time. Eight of these 18 visits were clearly related to prior medical conditions such as a chemotherapy-related encounters and were thus included in group 1. The other ten were deemed to be post-COVID-related encounters, five (5.1%) of whom were discharged from the ED (group 2) and five (5.1%) of whom were readmitted (group 3). Their clinical courses are described in Tables 2 and 3. Follow-up at 7 days was documented in 93.8% of patients (107/114); of these, three (2.8%) had a post-discharge ED visit within 7 days, all of whom were readmitted (Table 3).

**Table 2:** Clinical Course of Patients with ED Visit Within 30 Days Post-Discharge. Visit Deemed Potentially Related to Prior COVID Admission

Patient	Post-Discharge Emergency Department Visit
1	Day 8: Ankle swelling. Ultrasound negative for DVT; attributed to venous stasis
2	Day 21: Subjective dyspnea. Attributed to anxiety vs. viral bronchitis suggested by CXR.
3	Day 22: Chest pain. Laboratory workup negative. CTPA, TTE, EKG, CXR negative.
4	Day 23: Fall. Attributed to mechanical causes, advanced age, frailty.
5	Day 28: Cough. CTPA negative, attributed to mild asthma exacerbation.

*CXR* chest X-ray, *CTPA* computed tomography pulmonary angiography, *TTE* transthoracic echocardiogram, *EKG* electrocardiogram

**Table 3:** Clinical Course of Patients with Hospital Readmission Within 30 Days Post-Discharge. Readmission Deemed Potentially Related to Prior COVID Admission

Patient	Readmission and Clinical Course
1	Day 1: Dyspnea. Did not require oxygen. Discharged after 12 h. Attributed to anxiety.
2	Day 3: Dyspnea. Prior discharge was against medical advice. Discharged after 7 days.
3	Days 5: Diarrhea. Attributed to azithromycin. Discharged after 2 days.
4	Day 11: Aphasia. Neuroimaging negative. Attributed to atypical migraine. Discharged after 3 days.
5	Day 24: Cough. Attributed to viral bronchitis. Discharged after 1 day.

Four of 99 patients (4.04%) were febrile (*T* ≥ 38 °C) in the 24 h prior to discharge; none of these patients had an ED visit in the 30-day post-discharge period. Thirty-one of 99 patients (31.3%) were discharged on oxygen with ambulation or at rest; only one was readmitted within 30 days and was no longer requiring oxygen at that time.

**Figure 1:**
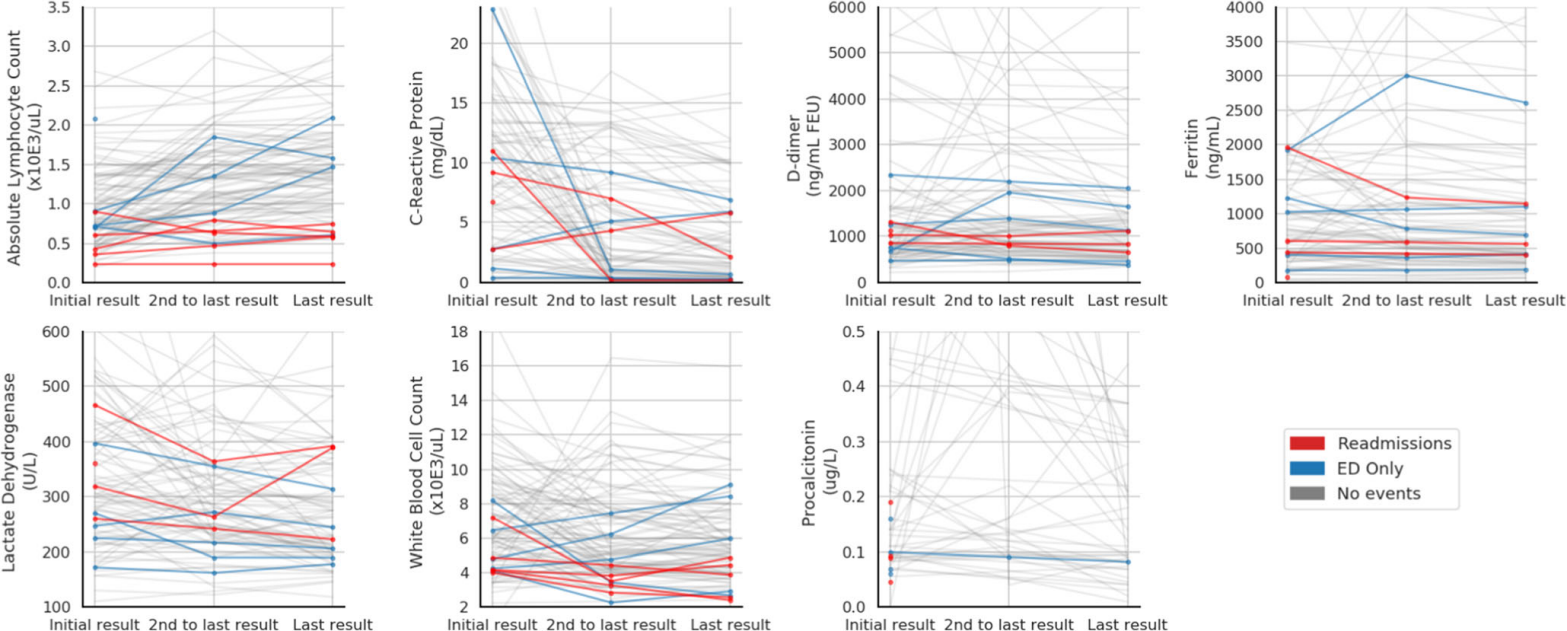
Post-discharge events and laboratory trends of inpatients with severe COVID-19.

Peri-discharge laboratory data are described in Table 4. Figure 1 visualizes the trajectory of multiple laboratory values on admission, during the hospitalization, and prior to discharge, showing no clear distinction in the pattern of these values among patients with and without post-discharge acute care needs. To confirm, inpatient clinical data around the time of discharge were analyzed for association with post-discharge outcomes. Each indicator was analyzed separately using a one-way ANCOVA with age and sex as covariates. None of the laboratory markers nor oxygen requirements were significantly associated with readmissions (CRP, *p* = 0.82; D-dimer, *p* = 0.58; ferritin, *p* = 0.58; LDH, *p* = 0.38; procalcitonin, *p* = 0.91; oxygen, *p* = 0.62; fever, not tested). WBC did not display a significant difference between the three groups (*p* = 0.10), and absolute lymphocyte count (ALC) values were significantly associated with post-discharge events (*p* = 0.004). Post hoc pairwise *T* tests using the Tukey HSD revealed that absolute lymphocyte counts among patients readmitted (mean 0.55 × 10E3/μL, standard deviation [SD] 0.19) were significantly lower than both patients who presented to the ED only (mean 1.57, SD 0.61) and patients who did not have a post-discharge encounter (mean 1.41, SD 0.55).

**Table 4:** Last Recorded Laboratory Values Prior to Discharge

	No Post-Discharge Encounter	Post-Discharge ED Visit	Readmission	*P* value
Patients	89	5	5	
Lymphocyte count (× 10E3/μL; mean (SD))	1.4 (0.5)	1.6 (0.6)	0.55 (0.2)	0.004
White blood cells (× 10E3/μL; mean (SD))	6.1 (2.5)	5.8 (3.0)	3.6 (1.1)	0.10
C-Reactive protein (mg/dL; mean (SD))	3.8 (4.0)	2.8 (3.3)	3.7 (3.1)	0.82
D-Dimer (ng/mL DDU; mean (SD))	1452.3 (1244.3)	1137 (727.7)	935.8 (227.2)	0.58
Ferritin (ng/mL; mean (SD))	885.8 (838.7)	1001.4 (964.5)	546.8 (446.7)	0.58
Lactate dehydrogenase (units/L; mean (SD))	289.3 (121.1)	226.4 (54.9)	341.3 (80.1)	0.38
Procalcitonin (μg/L; mean (SD))	0.35 (0.80)	0.30 (0.44)	0.22 (0.24)	0.91

## DISCUSSION

This report describes our experience with 99 patients hospitalized with COVID-19 at UCLA Medical Center. These patients were treated by hospitalist and critical care physicians, early on in the pandemic, at a time when concern for an inpatient census surge was high. Although our capacity for treating patients was never stressed, this concern spearheaded discussion of how to continually maximize inpatient capacity while prioritizing safe discharges. Little guidance was available to inform discharge criteria, so providers used their clinical judgment and shared decision-making with the patient to agree on a way forward. This created areas of clinical uncertainty, such as the proposition to discharge a patient who had a virus that was incompletely understood, on oxygen therapy. As such, close follow-up, either in-person or via telehealth, was arranged for these patients to ensure their continued clinical stability post-discharge. This allowed us to learn about any clinical deterioration after hospitalization and understand if peri-discharge clinical characteristics seemed to be associated with such cases.

In analyzing these patients’ peri- and post-discharge courses, we found that persistent fever, oxygen requirement, and laboratory abnormalities in patients hospitalized with COVID-19 were not associated with emergency room visits or readmissions within 30 days of discharge. To our knowledge, this is the first case series examining the relationship between inpatient clinical data and post-discharge courses of hospitalized adults with COVID-19. Our results shed light on multiple clinical questions and may assist with challenging clinical decision-making in the inpatient setting.

Some patients, despite being weaned off supplemental oxygen therapy, may still have markedly elevated and persistently uptrending inflammatory markers. An elevated D-dimer may persuade a clinician to postpone the discharge for fear of an undiagnosed thrombus. There is little data to guide providers about the appropriate course of action in this context. Our experience indicates that the trajectories of ferritin, D-dimer, LDH, CRP, and procalcitonin do not identify patients at risk of ED visits or readmissions within 30 days of discharge. This may suggest that, in the absence of other clinical findings that would caution against discharge, extending observation for patients with alarmingly abnormal laboratory markers is likely unnecessary in the setting of clinical improvement.

Other patients have a persistent low-level oxygen requirement despite their reports of resolving dyspnea and generally improved clinical condition, leaving clinicians unsure of the appropriateness of discharge. In this case series, 31.3% of patients were discharged on oxygen therapy, a common practice that is helping to relieve the strain on hospital capacity. Only one of these patients was readmitted within 30 days and was no longer requiring oxygen at that time. As such, it appears that discharging patients who still require supplemental oxygen, but who are otherwise clinically improving, does not appear to be associated with post-discharge need for acute care. This practice will likely continue to be an important component of post-hospitalization care for clinically stabilized inpatients with COVID-19, especially in the setting of an ongoing pandemic.

Similarly, some patients develop a fever on their planned day of discharge, in the setting of continued subjective improvement. Clinicians must ask themselves if, after a targeted workup, such patients should remain for further observation. In our study, only 4% of patients were febrile in the last 24 hours of hospitalization, potentially reflecting the clinician or patient’s hesitation toward discharge. None of these patients had an acute care need in the 30-day post-discharge period. Although limited by a small dataset, absent evidence of alternative causes of fever, physicians may feel more confident discharging a patient who is improving despite the persistence of fever. This is supported by reports revealing that a minority of patients with COVID-19 may continue to experience fevers 2 to 3 weeks after diagnosis.^19^ This case series calls for reconsideration of current recommended discharge criteria and encourages further studies to better understand the correlations between inpatient clinical factors and post-discharge acute care needs.

Notably, our data suggest an association between lymphopenia and post-discharge readmission within 30 days. While statistically significant, the sample size is very small, with only five patients requiring readmission. Furthermore, it would be clinically implausible to suggest that lymphopenia contributed to or predicted the types of complaints that these five patients re-presented with (Table 3), such as anxiety and antibiotic-induced diarrhea. As such, our limited data does not support the notion of persistent lymphopenia as a significant barrier to discharge in the setting of clinical improvement.

Given the paucity of standard clinical guidance and research on discharge criteria to date, a conservative approach favoring extended inpatient observation is likely frequently used. However, the downstream effects of this may be sizeable. First, this practice may unnecessarily limit inpatient bed availability for other acutely ill inpatients, which is of major importance in the setting of a pandemic. Second, prolonged length of stay increases risk of nosocomial infection, pressure ulcers, and deep vein thrombosis, and increases costs on healthcare systems. Finally, such a practice contributes to extended healthcare worker exposure to additional infected patients, unnecessarily increasing risk of hospital-acquired COVID-19. In hopes of foregoing these avoidable consequences, we offer this single-center experience as the first case series of its kind supporting reliance on clinical improvement rather than fixation over laboratory abnormalities, persistent oxygen requirement, or continued fever in the absence of other causes.

Our study has several limitations. First, this is a small, single-center case series, and as such is aimed only at providing a descriptive picture of the peri- and post-discharge courses of patients hospitalized with COVID-19. Larger, multi-center analyses are needed to augment and expand this dataset. Importantly, we acknowledge the significant proportion of discharged patients with persistent symptoms such as fatigue and dyspnea which can last for weeks to months after the acute illness subsides.^20^ Our study primarily serves to provide some reassurance to clinicians that, while a patient may remain symptomatic, abnormal laboratory results, oxygen requirement, or persistent fever do not seem to be, on their own, cause for prolonging the hospitalization, as 97.2% of patients did not require acute care within 7 days of discharge. Second, although every effort was made to ensure complete follow-up, it is possible that some patients sought acute care outside of the UCLA Health System and thus were not captured in the electronic health record or by the post-discharge clinical documentation. Third, patients included in this study were admitted before the efficacy of corticosteroids became clear for hypoxic patients with COVID-19, and results must be interpreted in light of any effect steroids may have on inflammatory markers in this new era of management. Last, our study did not examine whether these inpatient indices correlated with persistent symptoms in the post-discharge period; however, it is unlikely that extending the length of hospitalization to allow for further observation would ameliorate such sequelae. A future direction could include utilizing remote patient monitoring programs for patients with COVID-19 to allow for the collection of sizeable datasets elucidating the extent of patient symptoms or vital sign abnormalities during recovery.^21^

At a time when SARS-CoV-2 continues to cause outbreaks and overwhelm healthcare facilities worldwide, this case series offers urgent practical guidance to help decrease inpatient resource utilization while providing physicians and patients with peri-discharge clinical decision support.

## References

[1] Dong E, Du H, Gardner L (2020). An interactive web-based dashboard to track COVID-19 in real time. Lancet Infect Dis..

[2] Qin C, Zhou L, Hu Z (2020). Dysregulation of Immune Response in Patients With Coronavirus 2019 (COVID-19) in Wuhan, China. Clin Infect Dis..

[3] Mardani R, Ahmadi Vasmehjani A, Zali F (2020). Laboratory Parameters in Detection of COVID-19 Patients with Positive RT-PCR; a Diagnostic Accuracy Study. Arch Acad Emerg Med..

[4] Chen G, Wu D, Guo W (2020). Clinical and immunological features of severe and moderate coronavirus disease 2019. J Clin Invest..

[5] Henry BM, de Oliveira MHS, Benoit S, Plebani M, Lippi G (2020). Hematologic, biochemical and immune biomarker abnormalities associated with severe illness and mortality in coronavirus disease 2019 (COVID-19): a meta-analysis. Clin Chem Lab Med..

[6] Terpos E, Ntanasis-Stathopoulos I, Elalamy I (2020). Hematological findings and complications of COVID-19. Am J Hematol..

[7] Tan C, Huang Y, Shi F (2020). C-reactive protein correlates with computed tomographic findings and predicts severe COVID-19 early. J Med Virol..

[8] Deng Y, Liu W, Liu K (2020). Clinical characteristics of fatal and recovered cases of coronavirus disease 2019 in Wuhan, China: a retrospective study. Chin Med J (Engl)..

[9] Young BE, Ong SWX, Kalimuddin S (2020). Singapore 2019 Novel Coronavirus Outbreak Research Team. Epidemiologic Features and Clinical Course of Patients Infected With SARS-CoV-2 in Singapore. JAMA.

[10] Wu C, Chen X, Cai Y (2020). Risk Factors Associated With Acute Respiratory Distress Syndrome and Death in Patients With Coronavirus Disease 2019 Pneumonia in Wuhan, China. JAMA Intern Med..

[11] Guan WJ, Ni ZY, Hu Y (2020). China Medical Treatment Expert Group for Covid-19. Clinical Characteristics of Coronavirus Disease 2019 in China. N Engl J Med..

[12] Zhou F, Yu T, Du R (2020). Clinical course and risk factors for mortality of adult inpatients with COVID-19 in Wuhan, China: a retrospective cohort study. Lancet..

[13] Huang C, Wang Y, Li X (2020). Clinical features of patients infected with 2019 novel coronavirus in Wuhan, China. Lancet.

[14] Wang D, Hu B, Hu C (2020). Clinical Characteristics of 138 Hospitalized Patients With 2019 Novel Coronavirus-Infected Pneumonia in Wuhan, China. JAMA..

[15] Lippi G, Plebani M, Henry BM (2020). Thrombocytopenia is associated with severe coronavirus disease 2019 (COVID-19) infections: A meta-analysis. Clin Chim Acta..

[16] COVID-19 Treatment Guidelines Panel. Coronavirus Disease 2019 (COVID-19) Treatment Guidelines. National Institutes of Health. Available from: https://www.covid19treatmentguidelines.nih.gov/. Accessed 16 Sept 2020. 34003615

[17] Song JW, Zhang C, Fan X (2020). Immunological and inflammatory profiles in mild and severe cases of COVID-19. Nat Commun..

[18] Centers for Disease Control and Prevention. Discontinuation of Transmission-Based Precautions and Disposition of Patients with COVID-19 in Healthcare Settings (Interim Guidance). Available from: https://www.cdc.gov/coronavirus/2019-ncov/hcp/disposition-hospitalized-patients.html. Accessed 16 Sept 2020.

[19] Tenforde MW, Kim SS, Lindsell CJ (2020). IVY Network Investigators; CDC COVID-19 Response Team; IVY Network Investigators. Symptom Duration and Risk Factors for Delayed Return to Usual Health Among Outpatients with COVID-19 in a Multistate Health Care Systems Network - United States, March-June 2020. MMWR Morb Mortal Wkly Rep.

[20] Carfì A, Bernabei R, Landi F (2020). Gemelli Against COVID-19 Post-Acute Care Study Group. Persistent Symptoms in Patients After Acute COVID-19. JAMA..

[21] Annis T, Pleasants S, Hultman G (2020). Rapid implementation of a COVID-19 remote patient monitoring program. J Am Med Inform Assoc..

